# Phagocytosis of *Staphylococcus aureus* by Macrophages Exerts Cytoprotective Effects Manifested by the Upregulation of Antiapoptotic Factors

**DOI:** 10.1371/journal.pone.0005210

**Published:** 2009-04-21

**Authors:** Joanna Koziel, Agnieszka Maciag-Gudowska, Tomasz Mikolajczyk, Malgorzata Bzowska, Daniel E. Sturdevant, Adeline R. Whitney, Lindsey N. Shaw, Frank R. DeLeo, Jan Potempa

**Affiliations:** 1 Department of Microbiology, Faculty of Biochemistry, Biophysics and Biotechnology, Jagiellonian University, Krakow, Poland; 2 Department of Immunology, Faculty of Biochemistry, Biophysics and Biotechnology, Jagiellonian University, Krakow, Poland; 3 Department of Biology, University of South Florida, Tampa, Florida, United States of America; 4 Research Technologies Branch, Rocky Mountain Laboratories, National Institute of Allergy and Infectious Diseases, National Institutes of Health, Hamilton, Montana, United States of America; 5 Laboratory of Human Bacterial Pathogenesis, Rocky Mountain Laboratories, National Institute of Allergy and Infectious Diseases, National Institutes of Health, Hamilton, Montana, United States of America; 6 Department of Periodontics, Endodontics and Dental Hygiene, University of Louisville School of Dentistry, Louisville, Kentucky, United States of America; Columbia University, United States of America

## Abstract

It is becoming increasingly apparent that *Staphylococcus aureus* are able to survive engulfment by macrophages, and that the intracellular environment of these host cells, which is essential to innate host defenses against invading microorganisms, may in fact provide a refuge for staphylococcal survival and dissemination. Based on this, we postulated that *S. aureus* might induce cytoprotective mechanisms by changing gene expression profiles inside macrophages similar to obligate intracellular pathogens, such as *Mycobacterium tuberculosis*. To validate our hypothesis we first ascertained whether *S. aureus* infection could affect programmed cell death in human (hMDMs) and mouse (RAW 264.7) macrophages and, specifically, protect these cells against apoptosis. Our findings indicate that *S. aureus*-infected macrophages are more resistant to staurosporine-induced cell death than control cells, an effect partly mediated via the inhibition of cytochrome *c* release from mitochondria. Furthermore, transcriptome analysis of human monocyte-derived macrophages during *S. aureus* infection revealed a significant increase in the expression of antiapoptotic genes. This was confirmed by quantitative RT-PCR analysis of selected genes involved in mitochondria-dependent cell death, clearly showing overexpression of *BCL2* and *MCL1*. Cumulatively, the results of our experiments argue that *S. aureus* is able to induce a cytoprotective effect in macrophages derived from different mammal species, which can prevent host cell elimination, and thus allow intracellular bacterial survival. Ultimately, it is our contention that this process may contribute to the systemic dissemination of *S. aureus* infection.

## Introduction

*Staphylococcus aureus* is a major human pathogen causing significant morbidity and mortality due to both community- and hospital-acquired infections. This pathogen causes a variety of diseases, including impetigo, cellulitis, food poisoning, toxic shock syndrome, necrotizing pneumonia, and endocarditis [1], [2]. Localised *S. aureus* infections are often followed by bacterial invasion of the vascular system, leading to bacteraemia and sepsis. A large number of virulence factors are known to contribute to pathogenesis, e.g. surface proteins that support colonization of host tissues, invasins (hyaluronidase) and proteases that promote bacterial spreading, surface factors (protein A) that inhibit phagocytic engulfment or toxins (hemolysins, leukotoxin, exotoxins) that damage host-cell membranes [1], [3]. The treatment of *S. aureus* infections is increasingly problematic due to the high prevalence of multi-antibiotic resistant strains, such as methicillin-resistant *S. aureus* (MRSA) [4], and the emergence of glycopeptide-insensitive (GISA) [5] and vancomycin-resistant *S. aureus* strains (VRSA) [6].

For many years *S. aureus* was classified as a typical extracellular pathogen. However, recent experiments assessing invasion and the intracellular survival of *S. aureus* in endothelial and epithelial cells, and osteoblasts [7]–[9], suggests that such events may contribute to the persistence of *S. aureus* during infections such as endocarditis, bovine mastitis and osteomyelitis [10]. Moreover, it has long been known that professional phagocytes may serve as intracellular reservoirs of *S. aureus* [11]. In keeping with this idea, recent *in vitro* studies have confirmed high level resistance by *S. aureus* to neutrophil [12] and macrophage [13] mediated killing.

Professional phagocytes play a key role in host defence by recognizing, engulfing, and killing microorganisms; yet only a small group of pathogens can persist inside these cells, evading host defences. In mammals various intracellular pathogens have evolved strategies, including the modulation of programmed cell death (PCD), which favours their survival. One such strategy, utilized by some bacteria, viruses and parasites, involves the induction of apoptosis in immune effector cells like neutrophils and macrophages [14], [15]. This kind of strategy seems to be employed by *S. aureus* infections, not only in epithelial and endothelial cells, but also in neutrophils and monocytes [8], [9], [16]–[18]. One may argue, however, that apoptosis of infected cells constitutes part of host defense, limiting the dissemination of intracellular microorganisms by prompting the effective clearance of infected cells by resident and recruited phagocytes [19]. The converse of this strategy is the repression of programmed cell death in invaded host cells, allowing the pathogen to replicate and/or silently persist, whilst remaining invisible to the immune system [20], [21]. Well-known pathogens which employ this strategy include *Legionella pneumophila*, *Chlamydiae* spp., *Rickettsia rickettsii*, and *Neisseria gonorrhoeae* [22]. Within this category two divergent approaches are employed by pathogens to protect their intracellular niche. Obligate intracellular bacteria either activate cellular processes, which render the infected mammalian cells resistant to apoptotic stimuli, or directly interfere with the apoptotic apparatus [23].

Apoptosis is a form of programmed cell death that is highly regulated and consists of diverse upstream pathways for the transmission of extracellular death signals into intracellular events. One apoptosis pathway, referred to as extrinsic, involves apoptosis mediated by death receptors, such as CD95 (Fas) and tumor necrosis factor-α (TNF-α) receptors [24]. On the other hand, in the intrinsic apoptosis pathway, various proapoptotic signals converge at the mitochondrial level, provoking the release of cytochrome *c* from mitochondria into the cytosol [25]. In the cytosol, cytochrome *c* binds Apaf-1 and activates caspase-9, which in turn activates caspase-3. Activation of the executioner caspases leads to cleavage of a variety of target proteins with structural or regulatory functions, including poly(ADP-ribose) polymerase (PARP), protein kinase C, nuclear lamins, and others causing silent cell destruction from within. The intrinsic pathway is regulated by several proteins associated with the mitochondrial outer membrane. Whereas proteins of the Bcl-2 family (e.g., Bcl-2, Bcl-xL or Mcl-1) exert antiapoptotic activity, the Bax and Bak proteins stimulate cell death [26]. These antiapoptotic molecules prevent translocation of cytochrome *c* from the mitochondria, while dimerization of proapototic proteins results in cytochrome *c* release. Moreover, overexpression of antiapoptotic factors, such as Bcl-2 and Bfl-1, has been demonstrated to abrogate the function of proapoptotic proteins, and elicit a protective effect on host cells [27]. In contrast, overexpression of proapoptotic members of this family, including Bax, has been shown to induce apoptosis [28]. Therefore, the expression levels of either pro- or antiapoptotic factors plays an important role in determining the life-or-death decision of host cells. Previous studies have shown that macrophages respond to intracellular pathogens, e.g. *Mycobacterium tuberculosis* infection, by transient activation of host cell signal transduction pathways, which leads to alterations in gene expression, and the induction of cytoprotective mechanisms [29], [30].

A recent study by our group has shown that *S. aureus* can persist inside macrophages for several days without affecting the viability of these cells [13]. To analyze the strategies adopted by *S. aureus* for survival within human monocyte-derived macrophages, we investigated whether macrophages infection by *S. aureus in vitro* can affect (enhance or prevent) staurosporine-triggered apoptosis in host cells. We found that *S. aureus* is capable of protecting macrophages from staurosporine-induced apoptosis by preventing cytochrome *c* release and the associated subsequent activation of caspase-3. We also defined gene expression profiles of human monocyte-derived macrophages after *S. aureus* infection. Microarray analysis revealed a significant upregulation of antiapoptotic genes, especially those involved in mitochondrial pathways, and, in stark contrast, a decrease in expression of proapoptotic genes.

## Materials and Methods

### Cell culture and phagocytosis assay

Human monocyte-derived macrophages (hMDMs) were separated from fractions of peripheral blood mononuclear cells (PBMCs) obtained from the blood of healthy donors using a lymphocyte separation medium (LSM; PAA) density gradient [13]. Blood was obtained from the Red Cross (RC), Krakow, Poland. RC de-identified blood materials as appropriate for human subjects confidentiality assurance. Thus, the current manuscript adheres to appropriate exclusions from human subjects approval. PBMCs were seeded at concentrations of 2×10^7^ and 3×10^6^ cells/well into 6-well and 24-well plates, respectively, and cultured in RPMI1640 medium (PAA) supplemented with 10% heat-inactivated autologous human plasma, 2 mM L-glutamine, and 50 µg/mL gentamicin (Sigma) in a humidified atmosphere of 5% CO_2_. After 24 h, non-adherent cells were removed and adherent monocytes were differentiated to macrophages for 7–10 days, with fresh medium changes every second day. The hMDMs phenotype was evaluated by immunofluorescent staining for CD14 (DakoCytomation), CD16 (DakoCytomation), CD11b (Becton Dickinson), and CD209 (Becton Dickinson) of detached cells and subsequent flow cytometry analysis. The routine procedure used in our laboratory yields at least 90% cells positive for the first three antigens with less than 1% cells staining with anti-CD209 antibodies. The murine macrophage cell line RAW 264.7 was maintained in DMEM (PAA) supplemented with 5% fetal bovine serum (FBS; PAA). Macrophage infection was performed using the Newman strain of *S. aureus* (kindly provided by T. Foster), *Escherichia coli* or *Bacillus subtilis* (both from laboratory stocks). To exclude the possibility that cytoprotective effects were the result of unspecific cell activation, experiments using latex beads (1.1 µm; Sigma) and 20 ng/mL PMA (Sigma) to stimulate macrophages were conducted. Bacterial strains were stored and cultivated as described previously [13]. Heat treatment (80°C for one hour) was used to kill bacteria. The viability of live and heat-treated bacteria was routinely verified by plating dilutions on TSB agar and counting colonies to determine CFU/mL. Phagocytosis assays were carried out for 2 hours at 37°C at a multiplicity of infection (MOI) of 1∶50 (hMDMs) or 1∶5 (RAW 264.7). After that time cells were rinsed 4 times with ice-cold phosphate-buffered saline (PBS; PAA). Any remaining non-phagocytosed bacteria were killed by culturing in medium containing 50 µg/mL gentamicin for 24 h. The medium was then replaced with fresh media without antibiotics, and cultures were maintained for the desired time.

### Viability assays

After *S. aureus* phagocytosis and/or treatment with inducers of apoptosis, macrophage viability was examined by lactate dehydrogenase (LDH) release or 3-(4,5-dimethylthiazol-2-yl)-2,5-diphenyl tetrazolium bromide (MTT) reduction assays. The LDH release assay was performed using a CytoTox96 Non-Radioactive Lactate Dehydrogenase Cytotoxicity Assay kit (Promega). Infected and control hMDMs in a 24-well tissue culture plate (3×10^5^ cells per well) were treated with 1 µM staurosporine (STS; Sigma) added 24 h post-infection as a stimulator of apoptosis. Samples were then incubated for 24 h, followed by the removal of 200 µL of culture medium, and transferred to a 96-well, flat-bottom plate. The LDH substrate was added to each well and incubated for 30 min at 37°C in the dark. LDH activity in the medium, corresponding to macrophage necrosis, was measured as an absorbance at 490 nm using an ELISA plate reader (SpectraMax 250; Molecular Devices). Cytotoxicity was calculated with the formula: % cytotoxicity = (experimental value - low control)×100/(high control – low control), where the low control is assay medium and the high control is assay medium supplemented with 2% Triton X-100 plus cells to define the maximum LDH release. Spontaneous release was always found to be below 10% of the maximum release. The mitochondrial activity of control and *S. aureus*-infected hMDMs treated with apoptotic stimuli was determined by the ability of mitochondrial succinate dehydrogenase to convert MTT to the blue compound formazan. Briefly, 24 h after the phagocytosis of *S. aureus*, hMDMs were stimulated with STS for 24 hours. Subsequently, 0.5 mL of the MTT reagent (Sigma) dissolved in serum free RPMI1640 (0.5 mg/mL) was added to the cells, followed by incubation at 37°C for 2 hours. Any formed formazan crystals were dissolved by adding acidified isopropanol solution, and the absorbance of the solution was spectrophotometrically measured at a wavelength of 570 nm. Any increase or decrease in mitochondrial activity results in a concomitant change in the amount of formazan formed, indicating the degree of cytotoxicity caused by bacterial infection and/or proapoptotic treatment. All assays were performed in triplicate.

### Fluorescence microscopy

An early feature of apoptosis, the externalization of anionic phospholipid phosphatydylserine (PS), was assessed by annexin V binding to surface exposed PS using an annexin V-FITC kit (Bender Med Systems). Briefly, macrophages were incubated for up to 6 h after phagocytosis of *S. aureus*. At the time points indicated, 2×10^6^ cells were labeled with FITC-conjugated annexin V in 2% RPMI for 15 min at RT in darkness, and processed according to the manufacturer's protocol.

4′-6-Diamidino-2-phenylindole (DAPI; Sigma), which forms a fluorescent complex with natural double-stranded DNA, was used to stain the nuclei of hMDMs after *S. aureus* phagocytosis. At the time points indicated cells were washed with PBS and then incubated for 10 min on ice with PBS containing 0.1% Triton X-100. Permeabilized cells were then treated with 4% PBS buffered paraformaldehyde solution containing 10 µg/mL DAPI. The morphology of cell nuclei was examined at an excitation wavelength of 350 nm. Nuclei were considered to have a normal phenotype when glowing brightly and homogenously. Apoptotic nuclei were identified by condensed chromatin.

Propidium iodide (PI; Molecular Probes) staining was performed to assess the integrity of macrophage plasma membranes after phagocytosis of bacteria and/or treatment with inducers of apoptosis.

Analysis of phagocytosed, FITC-labeled *S. aureus* by macrophages was performed as follows. Bacterial samples, prepared as described in the cell culture section, were washed and resuspended in PBS containing FITC at a final concentration 100 µg/mL, followed by incubation for 30 min at 37°C. After incubation the bacteria were extensively washed and phagocytosis assays were performed as described previously. For those macrophages that engulfed *S. aureus* – FITC was observed using a Nikon Eclipse T*i* microscope.

### Analysis of caspase-3 activity

The activity of caspase-3, a main executioner protease involved in the apoptotic process, was determined by release of 7-amino-4-trifluoromethyl-coumarin (AFC) from a DEVD-AFC peptide substrate (Sigma). Cells (2×10^6^), both control and samples exposed to *S. aureus*, with or without apoptotic stimuli were collected by centrifugation (200×g, 5 min, 4°C), washed with ice-cold PBS and resuspended in 100 µL of lysis buffer (50 mM Tris, pH 7.5, 150 mM NaCl, 1% NP-40, 0.5% deoxycholic acid, 0.1% SDS). Samples were then incubated on ice for 20 min to lyse cells, with lysates recovered by centrifugation at 16,000×g for 10 min. The protein content of supernatants was measured using the BCA method. Caspase activity was determined by transferring aliquots of supernatant into buffer (40 mM Pipes, 20% sucrose, 200 mM NaCl, 0.2% CHAPS, 2 mM EDTA) containing DEVD-AFC and recording the increased fluorescence (λ_ex_ = 350 nm, λ_em_ = 460 nm) of any released AFC using a Spectra Max Gemini EM (Molecular Devices). Additionally, the cleavage of polyadenosine-diphosphate-ribose polymerase (PARP) from its native 116 kDa to its processed 85 kDa form by caspase-3 was determined from cellular pellets via Western blot.

### DNA fragmentation

Briefly, 2×10^6^ *S. aureus* and/or apoptotic, stimuli-treated or untreated macrophages were washed with cold PBS, harvested and collected by centrifugation (200×g, 5 min). Cells were then resuspended in 350 µL of lysis buffer (10 mM Tris pH 7.8, 5 mM EDTA, 0.5% SDS) and incubated at 65°C for 60 min. Lysates were then treated with RNaseA (30 µg/mL, 37°C, 1 h; Fermentas) and proteinase K (30 µg/mL, 50°C, 1 h; Fermentas) and extracted twice with an equal volume of phenol-chloroform. DNA was then precipitated at −20°C with 0.3 M sodium acetate −95% ethanol for 24 h. Precipitated DNA was harvested by centrifugation (13,000×g, 10 min, 4°C), washed with ice-cold 75% ethanol and dried. DNA was then dissolved in 30 µL of Tris-EDTA buffer and subjected to electrophoresis on a 2% agarose gel containing ethidium bromide (1 µg/mL) at 5 V/cm for 90 min. The analyzed DNA samples were compared with standard size fragments using a 1 kb DNA marker (Promega). DNA fragmentation and degradation was visualized by UV light and photographed.

### Flow cytometric analysis

Flow cytometric analysis was performed to assess changes in mitochondrial membrane potential (Δψ_m_) and to estimate the amount of mitochondria in *S. aureus*-treated hMDMs. To evaluate changes in ψ_m_, infected and control hMDMs were stained for 15 min at 37°C with 200 nM MitoTracker Red CMXRos (Invitrogen), a potential-sensitive fluorescent dye. After incubation cells were harvested and analyzed by a FACScan flow cytometer (Becton Dickinson) using the CellQuest software.

### Protein isolation and Immunoblotting

Whole cellular extracts from control and stimulated cells were prepared from cells detached using a rubber policeman, and harvested from culture medium into Eppendorf tubes. Cells were washed twice with PBS (250×g, 5 min), suspended in 100 µL of RIPA-lysis buffer (0.25% Na-deoxycholate, 0.5% Nonidet P-40, 0.05% SDS, protease inhibitor cocktail, 2.5 mM EDTA in PBS) and stored at −20°C.

For cytochrome *c* release and mitochondrial protein analysis, cells were fractionated into mitochondrial and cytoplasmic fractions. Briefly, 1×10^7^ hMDMs were scraped and collected by centrifugation (200×g, 5 min). Pellets were then washed twice with cold PBS, resuspended in buffer A (20 mM HEPES-KOH pH 7.4, 250 mM sucrose, 10 mM KCl, 1.5 mM Na-EGTA, 2 mM DTT, protease inhibitor cocktail) and incubated on ice for 20 min. Next, cells were disrupted by 30 circles of shearing through an insulin needle, followed by centrifugation (800×g, 10 min, 4°C). Mitochondria in the supernatant were pelleted by centrifugation (22,000×g, 15 min, 4°C). The resulting supernatant, representing the soluble cytoplasmic fraction, was frozen at −70°C, while the mitochondrial pellets were lysed with buffer B (50 mM HEPES pH 7.4, 1% Nonidet P-40, 10% glycerol, 1 mM EDTA, 2 mM DTT, protease inhibitor cocktail) during 20-min incubation on ice. Debris were removed by centrifugation (22,000×g, 15 min, 4°C) and the absence of cytoplasmic contamination was confirmed by the negligibly low level of the lactate dehydrogenase activity in this fraction. Supernatants containing mitochondrial proteins were stored at −70°C.

Equal amounts of protein from either whole cell extracts or subcellular fractions were separated using SDS-PAGE (8%, 12% or 16% gels depending on the molecular mass of the proteins of interest) and electrotransferred onto nitrocellulose membranes (Immobilon-PSQ; Millipore or nitrocellulose BioRad) in buffer composed of 25 mM Tris, 0.2 M glycine, 20% methanol (30 V, overnight). Membranes were stained with Ponceau S to analyze the efficiency of transfer and the equal loading of samples. Non-specific binding sites were blocked with 3% BSA in TTBS buffer (20 mM Tris, pH 7.5, 0.5 M NaCl, 0.05% Tween 20) for 1 h, followed by 1–2 hours incubation with the relevant primary antibody: 2,000-fold diluted anti-PARP (Biomol), 1,000-fold diluted anti-caspase-3 (Santa Cruz Biotechnology), 2,000-fold diluted anti-cytochrome *c* (BD Biosciences), 3,000-fold diluted anti-β-actin (Sigma) 2,000-fold diluted anti-COX IV (Molecular Probes), 1,000-fold diluted anti-cathepsin D (Calbiochem). Membranes were washed extensively in TTBS buffer and incubated with secondary horseradish peroxidase (HRP)-conjugated antibodies, donkey anti-rabbit IgG (Promega, 20,000-fold diluted or Amersham, 10,000-fold diluted) or sheep anti-mouse IgG (Sigma, 20,000-fold diluted), for 1 h in TTBS buffer containing 1% BSA. Membranes were washed (4×15 min) in TTBS buffer and blots were developed via ECL detection (Western Blotting Detection Reagents; Amersham Biosciences).

### Microarray analysis

Phagocytosis assays were performed using 2×10^6^ hMDMs as described previously. Control and *S. aureus*-exposed cells (8, 24 and 48 h post-infection) were washed twice with PBS and RNA was isolated using a RNeasy Mini Kit (Qiagen) according to the manufacturer's instructions. Microarray analysis was performed using HU133+2 human Affymetrix GeneChips as described earlier [31] but with modifications. Experiments were performed using hMDMs from 5 donors at each time point, using a separate GeneChip for each donor. Affymetrix GeneChip Operating Software (GCOS v1.4, http://www.affymetrix.com) was used to perform the preliminary analysis of the GeneChips. All GCOS *.*cel* files were scaled to a trimmed mean of 500 to produce the *.*chp* files. A pivot table with all samples was created and included calls, call p-value and signal intensity for each gene. The pivot table was then imported into GeneSpring GX 7.3 (http://www.chem.agilent.com). Hierarchical clustering (condition tree) using a Pearson correlation similarity measure with average linkage was used to produce a dendrogram that indicated biological replicates were grouping together. A separate analysis was prepared from the *.*cel* files by directly importing the files into Partek Genomics Suite software (Partek Inc. Saint Louis, Mo.) and running the quantile normalization to produce a principal component analysis (PCA) plot. An ANOVA was run from this normalization to produce p-values corrected for multiple comparisons using the false discovery rate (FDR). Significance Analysis of Microarrays (SAM) was also performed and these data were combined into a single custom Excel worksheet consisting of all statistical and quality filters to produce the final gene list. We initially defined genes as differentially expressed when changes in transcript levels were statistically significant by ANOVA and were at least 2-fold increased or decreased compared with unstimulated cells, and transcripts has to pass all quality filters. Complete microarray data are posted on the Gene Expression Omnibus (GEO, http://www.ncbi.nlm.nih.gov/geo/, accession number GSE13670).

### RNA extraction and Quantitative Real-Time PCR

Total RNA was extracted from cultured hMDMs using a RNeasy Mini Kit (Qiagen) according to the manufacturer's instructions. RNA samples were DNAse treated (Roche) and used for cDNA synthesis reactions in a total volume of 20 µL containing 0.5 µg of each RNA sample, 0.5 µg oligo (dT)_18_ primer, 20 U of RiboLock Ribonuclease Inhibitor, 1 mM dNTP mix and 40 U of M-MuLV Reverse Transcriptase (RevertAid™ First Strand cDNA Synthesis Kit; Fermentas). Quantitative PCR reactions were performed using a qPCR SYBR Green Kit (Finzymes) and a Rotor-Gene RG-3000 real-time thermocycler (Corbett Research), with a final reaction volume of 20 µL. The reaction mixes contained 1 µL of cDNA sample, 0.2 µM of each primer and a relevant fluorogenic probe. Forward and reverse primer sequences specific for *BCL2*, *BAX*, *MCL1* and the house keeping *EF2* gene are listed in **Table 1**. After 5 min of initial denaturation at 95°C, reactions were carried out at the following conditions: denaturation, 95°C for 20 sec; annealing, 62°C for 20 sec; extension, 72°C for 30 sec; followed by a final elongation step of 72°C for 20 min. Triplicate samples were analyzed for each reaction and the mean values calculated. Data analysis was conducted using the “delta-delta Ct” quantification method [32]. qPCR reaction products were resolved on non-denaturing 1.5% agarose gels and visualized by staining with ethidium bromide.

**Table 1:** Oligonucleotides used in this study.

Oligonucleotide	Sequence
*BAX*-F	5′-TGGCAGCTGACATGTTTTCTGAC
*BAX*-R	5′-TCTGGTCCCACCAACCCTGC
*BCL2*-F	5′-CAGATGCACCTGACGCCCTT
*BCL2*-R	5′-AGGTCCTATTGCCTCCGACCC
*MCL1*-F	5′-TAAGGACAAAACGGGACTGG
*MCL1*-R	5′-ACCAGCTCCTACTCCAGCAA
*EF2*-F	5′-TCAGCACACTGGCATAGAGGC
*EF2*-R	5′-GACATCACCAAGGGTGTGCAG

### Statistics

Results were analyzed for statistical significance using the Student's *t*-test. Differences were considered significant when p<0.05.

## Results

### *S. aureus* induces an incomplete activation of apoptosis during intracellular survival in hMDMs

**Figure 1:**
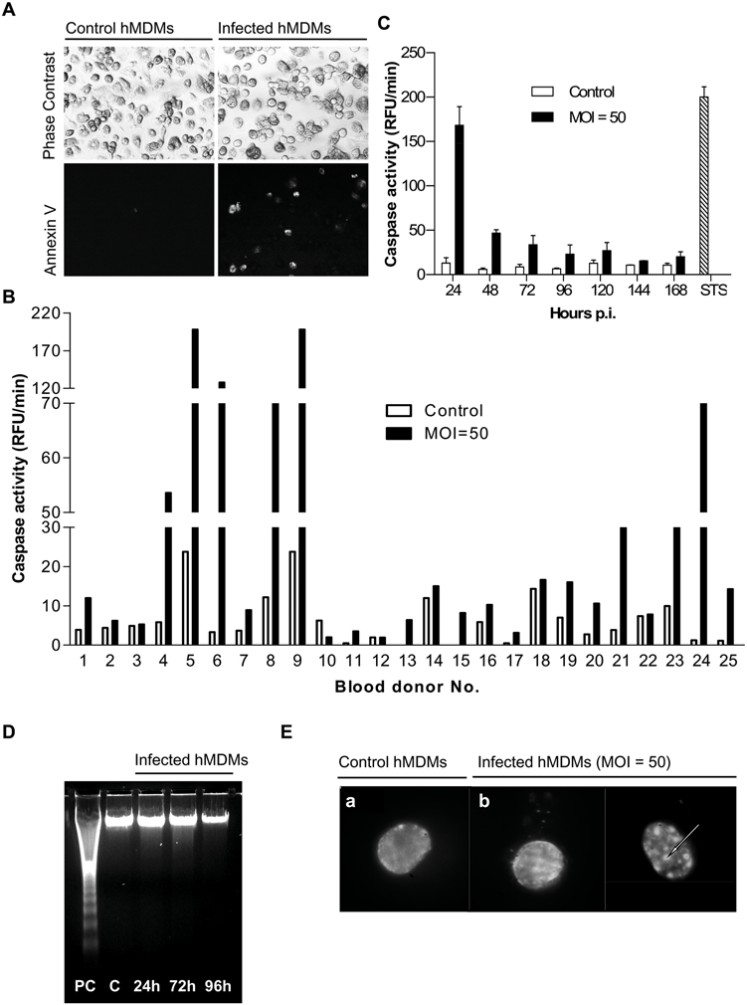
*S. aureus* infection of hMDMs causes transient caspase-3 activation in a donor-dependent manner without development of late apoptotic features. hMDMs were allowed to phagocytose *S. aureus* at a ratio of 50 bacteria per macrophage (MOI 1∶50) for 2 h. Bacteria were then removed and cells were cultured for an additional 6–168 h. At the indicated time points the infected cells were analyzed for apoptotic changes. (A) Phosphatidylserine (PS) externalization to the cell surface was examined under a fluorescence microscope after staining cells with FITC-annexin V. Upper and lower panels show transmission and fluorescent light micrographs (×20), respectively, of control, mock-infected macrophages (left panel) and *S. aureus*-infected cells (right panel) maintained in culture for 6 h. The presented photographs are representative of a minimum of 20 fields observed during 3 independent experiments. (B) The activity of caspase-3 (RFU/min) in infected and control cultures of hMDMs originating from 25 blood donors was measured with DEVD-AFC 24 h after *S. aureus* phagocytosis. (C) Infected macrophage cultures were maintained for 168 h post-phagocytosis and caspase-3 activity (RFU/min) was determined at 24 h intervals. The figure is representative of several experiments, each performed in triplicate, using hMDMs cultures responding to *S. aureus* infection with strong procaspase-3 activation. Macrophages treated with STS for 24 h were used as a positive control. STS, staurosporine. (D) Lack of DNA fragmentation examined by agarose electrophoresis at 24, 48 and 72 h after *S. aureus* phagocytosis (representative result). (E) Assessment of chromatin condensation in infected hMDMs (24 h post-phagocytosis) using DAPI staining fluorescence microscopy (100×). Only occasional, slight chromatin condensation was visible (arrowheads). The results presented are from one representative experiment out of 5. Scale bars = 10 µm.

Macrophages are fairly resistant to apoptosis, as interaction with some bacterial pathogens and/or their toxins induces only limited apoptosis in these cells [21], [33]. In keeping with this finding, we have recently shown that the phagocytosis of *S. aureus* by hMDMs leads to a prolonged intracellular survival of this pathogen in phagocytes without affecting the viability of host cells [13]; despite a transient increase in caspase-3 activity in the infected cells. To explore this observation further hMDMs were infected with *S. aureus* strain Newman, and, at different time points, a variety of tests to detect apoptosis were conducted. Cells treated identically, but without *S. aureus*, were also analyzed in all experiments as a control (mock-infected cells). In most analyses 1 µM staurosporine was used as a positive control for apoptotic induction. Macrophages infected with *S. aureus* and control cells were stained with FITC-conjugated annexin V to detect an early event in apoptosis, the exposure of phosphatidylserine on the cell surface. In contrast to mock-infections, in which the appearance of apoptotic cells was sporadic (2.1±0.65%), the phagocytosis of *S. aureus* increased the number of annexin-positive/PI-negative cells by up to 20% of total cell counts (15.5±3.25%) (**Fig. 1A**). *S. aureus* uptake was associated with the activation of caspase-3 only in a subset of cultures. Out of 25 hMDMs cultures from different donors, the robust activity of caspase-3 (100–250 RFU/min) was observed only in 3 cases (12%), whilst in 9 cases (36%) macrophages responded with a moderate increase in activity (10–80 RFU/min). In the majority of infected hMDMs cultures, no increase in caspase-3 activity was observed 24 h post-phagocytosis, when compare to control cells (**Fig. 1B**). Moreover, infected *in vitro* individual hMDMs cultures exhibited very similar bacterial load (CFU in cell lysates) in consecutive days post-phagocytosis. This strongly suggests that diverse level of caspase-3 activity is donor-dependent. Using hMDMs from selected donors we determined changes in caspase-3 activity during prolonged *S. aureus* infection over the course of 168 h. In all cases of significant, *S. aureus*-triggered caspase-3 activation, the maximal activity occurred at 24 h post-phagocytosis, and then decreased to control levels (**Fig. 1C**). In these cultures caspase-3 activity was correlated with the accumulation of a signature PARP fragment (p85), resulting from PARP cleavage by caspase-3 (data not shown). Nevertheless, despite this clear apoptotic pathway activation feature (very high activation of caspase-3), no hallmarks of late apoptotic changes, such as DNA fragmentation, were observed in infected hMDMs (**Fig. 1D**). The DNA integrity of infected cells was also confirmed by negative results from the TUNEL assay, which visualizes fragmented chromosomal DNA from apoptotic cells via the incorporation of fluorescein-12-dUTP at its 3-ends (data not shown). Furthermore, morphological analysis of DAPI-stained cells only occasionally revealed slightly condensated nuclei 4±0.5% (**Fig. 1E**, arrow) at 24 h post-phagocytosis. In all cases, the presence of significant numbers of apoptotic nuclei was undetectable throughout the infection. Taken together our results indicate that *S. aureus* is able to induce the initial steps of apoptosis, including phosphatidylserine exposure, decrease of mitochondrial potential, release of cytochrome *c* and caspase-3 activation, in donor-dependent manner; yet this does not lead to finalization of the apoptotic process (no oligonucleosomal DNA fragmentation or secondary necrosis features). With respect to the level (high or low) of apoptotic response hMDMs obtained from individual donors can be divided into two general groups: susceptible and fairly resistant (majority) to *S. aureus*-induced apoptosis. In all experiments described below we routinely use the later group of hMDMs as more representative. Furthermore, most interesting results were verified using the RAW 264.7 cell line.

### Infection of macrophages with *S. aureus* protects cells from STS-induced cell death

**Figure 2:**
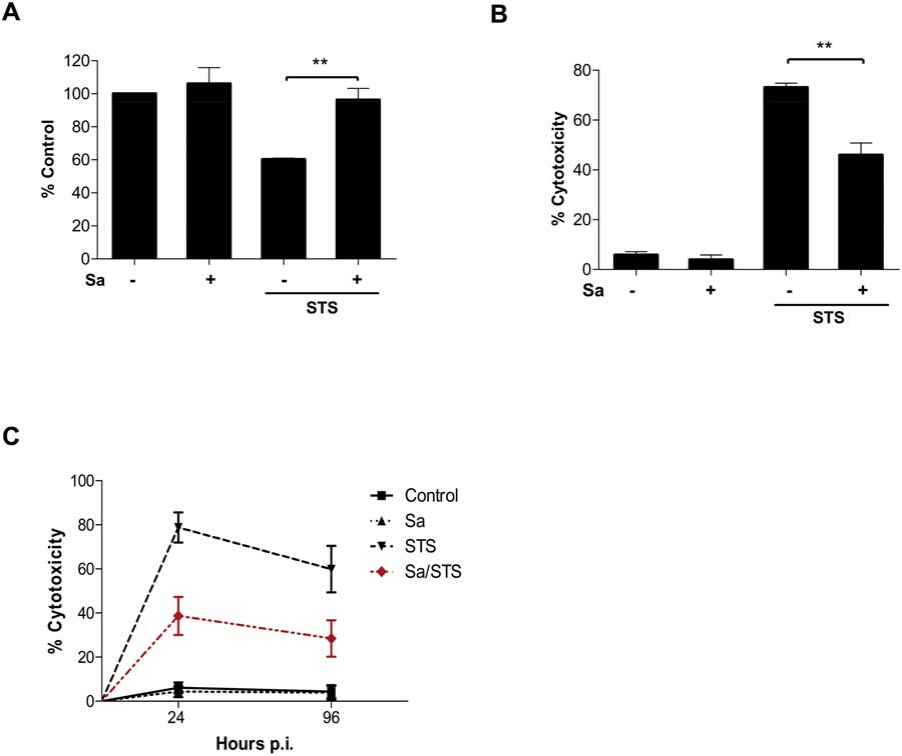
*S. aureus*-infected hMDMs show a decreased susceptibility to the cytotoxic effects of STS. Control and *S. aureus*-infected cells (24 h post-phagocytosis) were treated with STS at a concentration of 1 µM for 24 h and cell viability was evaluated by MTT (A) and LDH (B) assays. (A) The mitochondrial metabolic activity is presented as the percentage of control hMDMs, which were considered to be 100%. (B) Plasma membrane permeabilization or cell lysis induced in mock- and *S. aureus-*infected hMDMs was determined as LDH activity levels. The experimental value was the LDH activity in the conditioned medium from the control and infected cells after STS stimulation for 24 h. The data shown is representative of at least three separate experiments, performed in triplicate, using hMDMs derived from different donors. **, p<0.01. (C) The cytotoxicity measured as the LDH activity levels in the conditioned medium from the mock- and *S. aureus-*infected hMDMs (24 and 96 h p.i.) after treatment with STS for 24 h. The data shown is representative of at least three separate experiments, performed in triplicate, using hMDMs derived from different donors. **, p<0.01.

To evaluate whether infection with *S. aureus* is able to inhibit cell death, human (hMDMs) and mouse macrophages (RAW 264.7), of both infected and control cells, were treated with staurosporine, a potent inducer of apoptosis commonly used in a macrophage model applied to study cytoprotective effect of various bacteria [20], [34]. As expected, staurosporine, at a concentration of 1 µM, efficiently induced control hMDMs cell death, as revealed by MTT and LDH cytotoxicity assays (**Fig. 2A and 2B**, respectively). Conversely, *S. aureus* phagocytosis had no effect on the reduction of tetrazolium (MTT) into its insoluble formazan product, nor did it cause the release of LDH. Moreover, even at the highest levels of infection there was no significant cleavage of MTT. Notably, *S. aureus*-infected hMDMs were significantly protected against the lethal effects of STS. Indeed, they retained 100% of their mitochondrial activity which was reduced to 60% in mock-infected cells treated with STS (**Fig. 2A**). In addition, a much higher percentage of infected cells treated with STS maintained plasma membrane integrity than similarly treated control macrophages, as indicated by lactate dehydrogenase (LDH) activity in culture media samples (**Fig. 2B**). This cytoprotective effect of hMDM STS-induced cell death was observed all the way up to 96 h post-infection (data not shown). The effect was maximal at 24 h and somehow less profound but still significant at the later stages of infection (**Fig. 2C**).

**Figure 3:**
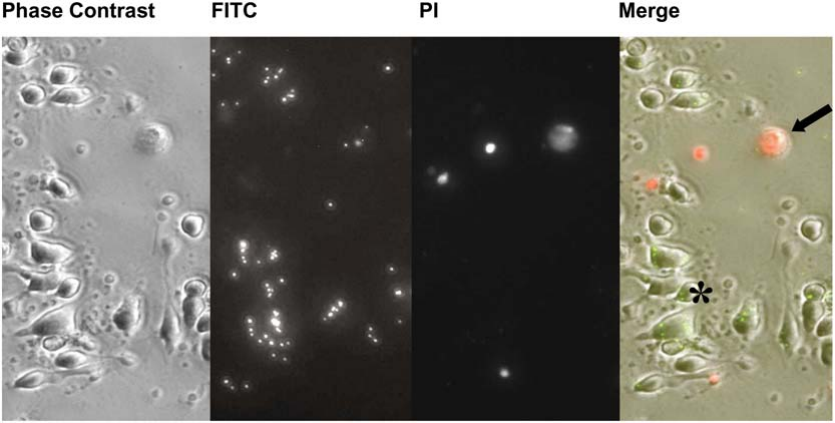
The intracellular presence of *S. aureus* protects macrophages (RAW 264.7) from STS-induced cell death. Macrophages were allowed to phagocytose FITC-labeled *S. aureus* at a ratio of 5 bacteria per macrophage (MOI = 1∶5) for 2 h. Extracellular bacteria were removed and cells were cultured in media with antibiotic for an additional 24 h, before being treated with STS (1 µM) in fresh culture medium for 3 h. Subsequently, cell viability (PI staining), as well as the presence of intracellular bacteria, were analyzed using a fluorescence microscope. (The figure presents transmission and fluorescent light micrographs of macrophage cultures infected with FITC-labeled *S. aureus* and treated with STS. The merged panel represents PI stained dead uninfected cells (arrows); and viable macrophages with intracellular *S. aureus* which maintained their cell membrane integrity (asterisks). The figure shows representative results of three independent experiments.

*S. aureus* phagocytosis leads to infection of only a subset of macrophages (50–70%) from a given population, and it can be anticipated that cells free of bacteria would be predominantly susceptible to STS. Therefore this effect would significantly mask the protective effect exerted by infection (**Fig. 2**). To verify this notion, and to determine the infection and cell vitality status of individual macrophages, we performed fluorescence microscopy analysis. Macrophages (RAW 264.7) were infected with FITC-labeled *S. aureus*, and, after treatment with STS, counterstained with propidium iodide. Notably, *S. aureus*-infected cells (green positive staining, asterisk) had anadherent morphology, and an intact cell membrane (PI negative staining), whereas the majority of non-infected cells had leaky membranes (PI positive staining) and a non-adherent morphology indicating cell death (**Fig. 3**, merged; **Table 2**). Collectively our data strongly argues that infection with *S. aureus* reduces the sensitivity of macrophages to staurosporine-induced apoptosis.

**Table 2:** Cell counts of viability assay experiments.

Experiment No.	% of PI-negative (live cells) containing intracellular *S. aureus*	% of PI-positive (dead cells) containing intracellular *S. aureus*
1	52.0	9.0
2	75.2	7.6
3	55.7	0.0
Mean±SD	61.0±12.4	5.5±4.8

Mouse macrophages infected with FITC-labeled *S. aureus* were treated with STS for 24 h, stained with PI and visualized by fluorescence microscopy. The table shows representative results of three independent experiments.

### *S. aureus* infection prevents STS and butyric acid-induced activation of caspase-3 in macrophages

**Figure 4:**
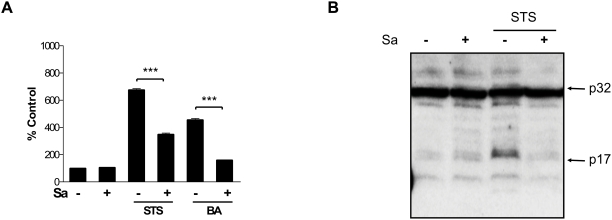
hMDMs infection with *S. aureus* inhibits caspase-3 activation induced by STS and butyric acid. (A) The effect of *S. aureus* infection on caspase-3 activity was measured with DEVD-AFC as a substrate in hMDMs after 24 h stimulation with STS or BA. The diagram is a representative result of an experiment performed in triplicate using macrophages isolated from a single donor. Bars represent mean±SD of caspase-3 activity (RFU/min). The caspase-3 activity of mock-infected cells was regarded as 100%. (B) Inhibition of procaspase-3 processing induced by STS in *S. aureus*-infected hMDMs. Macrophages with or without *S. aureus* infection were STS treated, and 24 h post-infection cells were lysed for Western Blot analysis using antibodies against caspase-3. Caspase-3 antibody staining was developed with a secondary antibody conjugated to horseradish peroxidase followed by visualization using ECL as described in the Materials and Methods.

To further analyze mechanisms hindering the cell death of macrophages infected with *S. aureus*, we determined whether apoptosis inhibition takes place upstream or downstream of caspase-3 activation. To this end we evaluated caspase activity in human and mouse macrophages upon induction of apoptosis by treatment with STS and butyric acid, as well as Fas ligation by specific antibodies. As expected, in the non-infected control cells (hMDMs and RAW 264.7) both STS and butyric acid induced a very high level of caspase-3 activity in contrast to anti-Fas antibodies, which exerted only a minor effect. Therefore, we used STS for all subsequent experiments. Significantly lower levels were induced in cultures infected with *S. aureus*, with 59.3%±8.4 and 45.3%±7.1 of control cell activity (treated with STS or butyric acid, respectively) observed (**Fig. 4A**).

To further confirm that caspase-3 activation is hindered in *S. aureus*-infected macrophages we performed Western Blot analysis of cell extracts from cultures treated with STS to detect active forms of caspase-3. In stark contrast to STS-exposed non-infected hMDMs, which presented a strongly immunoreactive band corresponding to active caspase-3 (p17), such a band was completely absent in control and *S. aureus* infected cultures, including those treated with STS (**Fig. 4B**).

**Figure 5:**
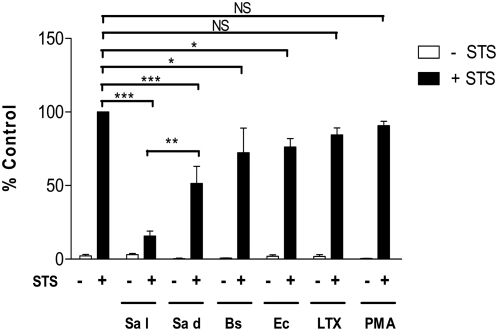
Inhibition of caspase-3 activation induced by STS in RAW 264.7 macrophages is dependent on *S. aureus* phagocytosis. The effect of live (Sa l) and heat-killed (Sa d) *S. aureus*, *B. subtilis* (Bs), *E. coli* (Ec) and latex beads (LTX) phagocytosis (MOI = 1∶5) on STS-induced caspase-3 activity in RAW 264.7. After phagocytosis cells were cultured for 24 h and treated with STS for 3 h. Caspase-3 activity was measured from cell lysates using DEVD-AFC as a substrate. STS-induced activity in control, resting cells was taken as 100%. Data are the mean±SD values from three independent experiments, each performed at least twice. *p<0.05; ***, p<0.001 significance as indicated in the figure.

Taking into account that the inhibition of procaspase-3 activation may have resulted from general phagocytosis-induced macrophage activation, we investigated if the observed effect was specific for live *S. aureus* uptake. Thus, we compared caspase-3 activity in PMA-treated murine macrophages and macrophages which had phagocytosed *B. subtilis*, *E. coli* live or heat-killed *S. aureus*, or latex beads (each of them at the same MOI = 1∶5); with each sample being subsequently treated with STS. Engulfment of these particles (bacteria or beads) alone did not cause any significant procaspase-3 activation 24 h post-phagocytosis. The proapoptotic effect of STS, measured as caspase-3 activation, was maximal in control, resting cells; whilst in cultures engaged previously in phagocytosis, caspase-3 activity was diminished (**Fig. 5**). In contrast to treatment with PMA and the uptake of latex beads, *E. coli* or *B. subtilis*, which only slightly affected procaspase-3 activation, the phagocytosis of *S. aureus* caused a significant decrease in caspase-3 activity. Notably, the most profound effect was exerted by the ingestion of live, rather than dead, *S. aureus* cells. Indeed this was found to be at only 15.6%±3.3 of those levels observed for control, resting cells treated with STS. This is in clear contrast to the effects exerted after the engulfment of dead bacteria, which only partially reduced caspase-3 activation (55.4%±11.6 of control value) in response to STS.

Taken together, these data suggest that intracellular infection with *S. aureus* may inhibit a key step in the mitochondrial pathway required for apoptosis. To our knowledge, such a profound inhibition of cell death in *S. aureus*-infected macrophages is an as yet undocumented event in the *in vitro* model system and prompted us to elucidate the mechanism(s) of this phenomenon.

### *S. aureus* suppresses STS-induced apoptosis in hMDMs by preventing the release of cytochrome *c* from mitochondria

**Figure 6:**
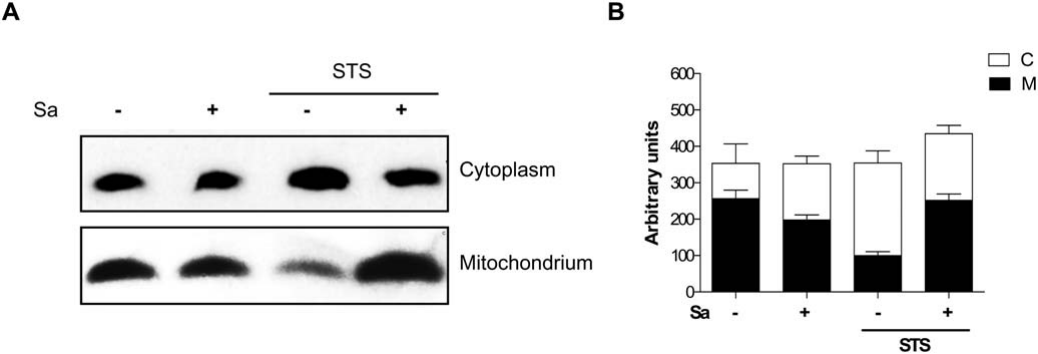
The infection of hMDMs with *S. aureus* prevents STS-stimulated cytochrome *c* release into the cytoplasm. Infected and control hMDMs were incubated with STS for 3 h and mitochondrial and cytosolic fractions were prepared as described in the Materials and Methods. (A) A representative immunoblot selected from four separate experiments. Cytochrome *c* was visualized by Western Blotting using anti-cytochrome *c* specific antibodies. Purity of fractions was ∼70% as assessed by anti-β-actin and anti-COX IV antibody (data not shown). (B) Individual band intensity acquired by blot scanning and expressed in arbitrary units.

The release of mitochondrial cytochrome *c* into the cytosol, and the activation of caspase-3, have both been reported as playing an essential role in cellular apoptosis [25]. To investigate whether the STS-induced release of cytochrome *c* is blocked in *S. aureus*-infected hMDMs, the amount of cytochrome *c* in cytosolic and mitochondrial fractions was analyzed via semiquantitative Western blot analysis. The purity of the mitochondrial and cytoplasmic fractions was tested by western blotting with an antibodies directed against cytochrome *c* oxidase subunit IV (COX IV), β-actin, and cathepsin D as mitochondrial, cytoplasmic and lysosomal marker, respectively. We found mitochondrial fraction basically free of cytoplasmic and lysosomal contamination. By direct comparison of the intensity of a cytochrome *c*-specific band in mitochondria-enriched and cytosolic fractions, we found that in control hMDMs most of the cytochrome *c* was present in mitochondria-enriched fractions (**Fig. 6**). The phagocytosis of *S. aureus* only slightly affected the balance between mitochondrial and cytoplasmic cytochrome *c* content (**Fig. 6B**). In stark contrast however, STS-treatment of uninfected, resting macrophages caused release of mitochondrial cytochrome *c* into the cytoplasm, with the majority of cytochrome *c* found in the cytosolic fraction. Significantly, this cytochrome *c* release was blocked to control levels if STS-treated cells were infected with *S. aureus* (**Fig. 6A, B**). These data argue that the presence of intracellular *S. aureus* within macrophages attenuates the propapototic effect of STS by preventing STS-induced release of cytochrome *c* from mitochondria.

Mitochondria play a central role in the control of the apoptotic process. By opening permeability transition pores in the inner mitochondrial membrane, membrane potential (Ψ_M_) rapidly dissipates. This process is considered to be a major determinant of the cellular commitment to death [35]. To examine whether the infection of hMDMs with *S. aureus* affects the loss of the mitochondrial potential induced by STS, cells were loaded with Mito-Tracker Red CMXRos at various time points after STS stimulation and the ΔΨ_M_ was analyzed by flow cytometry.

**Figure 7:**
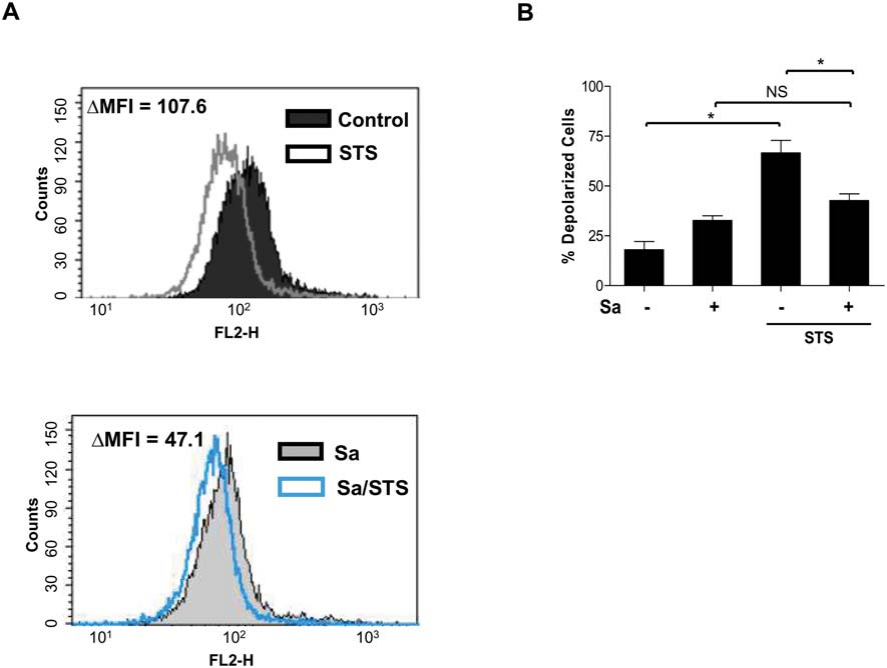
*S. aureus* phagocytosis partially protects infected hMDMs from an STS-induced drop in mitochondrial membrane potential. (A) Control and *S. aureus*-infected cells were stimulated with STS for 3 hours. Subsequently the cells were stained with CMXRos and subjected to flow cytometry analysis as described in the Materials and Methods. Representative histograms are shown. (B) The number of cells with depolarized mitochondrial membranes was determined. The diagram illustrates a result of several performed experiments using hMDMs derived from different donors. Bars represent the mean amount of depolarized cells calculated as % of the total analyzed cells ±SD; *, p<0.05; NS, not significant.

The infection of macrophages (derived from individual donors) by *S. aureus* caused, in the majority of cases (62.5%), only a small, statistically insignificant decrease in the mean fluorescence intensity (MFI), a measure of Ψ_M_, in comparison to control mock-infected cells (**Fig. 7**). Nevertheless, in hMDMs cultures derived from (37.5%) donors, *S. aureus* infection significantly lowered Ψ_M_ 24 h post-phagocytosis. The drop of MFI correlated well with the increased level of cytochrome *c* in the cytoplasm (data not shown) and the activation of caspase-3 in these cultures (**Fig. 1B**). Despite these variations, in all of the investigated cultures, staphylococcal infection radically protected the STS-induced decline of Ψ_M_, as illustrated by representative examples shown in **Fig. 7B**. Collectively, these data suggests that *S. aureus* induces an antiapoptotic program by stabilizing Ψ_M_, blocking cytochrome *c* release and subsequent caspase-3 activation.

### *S. aureus* infection induces the expression of antiapoptotic regulators in hMDMs

It has previously been described that bacterial pathogens may regulate cell death via the modulation of host cell gene expression, in order to prolong their intracellular survival [36]–[38]. Therefore, based on our observation that *S. aureus* induces cytoprotective mechanisms within macrophages, we analysed the gene expression profiles of human monocyte-derived macrophages 8, 24 and 48 h after *S. aureus* phagocytosis. Analysis of the changes in gene expression, evaluated in five independent microarray experiments in hMDMs derived from separate individuals, indicated that whereas the level of control transcripts, including *18S rRNA*, was unaffected, a significant increase in the expression of antiapoptotic genes was observed (**Table 3**). Interestingly, the upregulation of these antiapoptotic genes was accompanied by a simultaneous downregulation in the expression status of some proapoptotic determinants (**Table 3**).

**Table 3:** Selected genes involved in apoptotic cell death with altered expression observed in hMDMs 8, 24 and 48 h after *S. aureus* phagocytosis.

Entrez Gene Anti-apoptosis	8 h	24 h	48 h	Ref.
*TNFAIP3* *	2.0	3.0/3.1	3.0/3.0	[53]
*CD40* *	4.4/2.9/3.3	5.5/3.2/3.6	2.6	[54]
*CFLAR* *	2.2/3.1/3.7/3.4/2.5	2.2/2.6/3.7/3.9/2.3	2.0/2.1/2.3/2.5	[55]
*NLRP3* *		2.1/2.1		[56]
*GIMAP5* *	2.6/2.4	2.2		[57]
*NFκB1*	2.2			[58]
*NFκB2* *	2.3/2.0	2.1/2.1		[58]
*VEGFA* *	4.7/10.9/3.7	4.9/9.3/4.1/5.7	2.8/5.2	[59]
*MCL1* *	2.1/3.4	2.2/2.5/4.1	2.0	[60]
*BCL3*		2.0		[61]
*STAT3* *	2.3/2.1/2.5	2.1		[62]
*STAT4*	6.0	6.3	3.5	[62]
*BIRC3*	3.3	3.5	3.1	[63]
*TRAF1* *	2.9/3.2	3.2/3.8	2.6/2.7	[64]

Pro-apoptosis	8 h	24 h	48 h	Ref.
*RHOBTB2*	−2.7	−2.3		[65]
*TNFRSF21*		−2.3/−2.6		[66]
*CASP1*	3.0	5.6/3.3/3.9		[67]
*CASP4*	2.3	3.0		[68]
*TNFRSF 10A*	2.3			[66]
*TNFSF 10*	37.2/31.1/33.3			[66]
*TRADD*	3.3/2.5			[69]

Anti-/Pro-apoptosis	8 h	24 h	48 h	Ref.
*TNF*	11	21.6		[70]
*STAT1*	3.3	5.5/2.6	2.4	[62]
*STAT2*	3.8/5.1	2.6/3.1		[62]
*CASP1*	3.0	5.6/3.3/3.9		[67], [70]
*CASP10*	2.9			[71], [72]

Data given are the mean fold-change from five independent samples using Affymetrix microarrays that compare transcript levels in uninfected and *S. aureus*-infected hMDMs.

* , genes represented by more then one probeset on the microarray.

**Figure 8:**
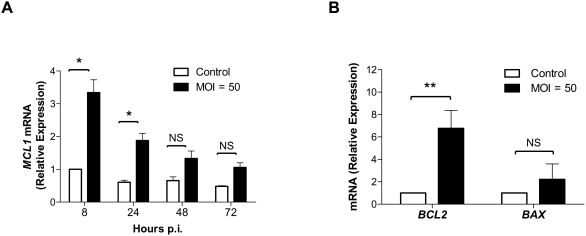
Staphylococcal infection of hMDMs results in changes in expression of apoptosis-related genes. (A) Upregulation of the antiapoptotic *MCL1* gene in hMDMs. The expression level of *MCL1* in uninfected and *S. aureus*-infected macrophages was monitored by quantitative real-time RT-PCR as described in the Materials and Methods. Data given are the mean values calculated from the results of three independent real time reactions. (B) Relative changes in mRNA expression of *BCL2* and *BAX* 8 h after *S. aureus* phagocytosis by macrophages. The data shown is representative of three separate experiments, performed in triplicate, using hMDMs derived from different donors. Bars represent mean relative expression ±SD; *, p<0.05; **, p<0.01; NS, not significant.

Since *S. aureus* apparently blocks apoptosis in macrophages upstream of the release of mitochondrial cytochrome *c*, we investigated the possible mechanism of this inhibition. To this end, we compared the expression levels of genes strictly involved in the mitochondrial cell death pathway. Microarray results revealed that from this group of genes, only *MCL1* was significantly affected by *S. aureus* phagocytosis. To verify changes in the *MCL1* expression in more specific terms, quantitative real time RT-PCR was carried out to measure the level of gene expression at different time points post-phagocytosis. As calculated based on the reference gene *EF-2*, an internal control whose expression was stable under all conditions tested, *MCL1* expression increased approximately 4-fold in cells 8 h after *S. aureus* phagocytosis (**Fig. 8A**). The elevated expression of *MCL1* decreased during the course of infection, yet at 72 h it was still 2-fold higher than that in mock-infected control cells (**Fig. 8A**). As a negative control, reverse transcriptase was excluded from the cDNA synthesis reaction to control for possible DNA contamination, and no amplification was observed (data not shown).

The microarray analysis revealed no significant changes in the expression levels of either *BCL2* or *BAX*. Nevertheless, because these genes are crucial in the regulation of mitochondrial membrane permeability we examined their expression by quantitative RT-PCR. This analysis revealed substantial upregulation of *BCL2*, but a small and statistically insignificant increase in *BAX* expression (**Fig. 8B**) in hMDMs 8 h after *S. aureus* phagocytosis.

Taken together, the combined results of our microarray and quantitative RT-PCR analyses provides strong evidence that the infection of monocyte-derived macrophages with *S. aureus* upregulates the transcription of antiapoptotic genes, which likely explains the observed restricted release of cytochrome *c* from mitochondria upon STS treatment.

## Discussion

In a recent *in vitro* study by our group we have shown that *S. aureus* phagocytosed by human monocyte-derived macrophages (hMDMs) can survive intracellularly for 4–7 days without affecting host cell viability. This proceeds until the cells are suddenly lysed by escaping bacteria, which then go on to proliferate to high numbers [13]. In the present study we have further investigated this phenomenon and have shown that the viability of infected cells is maintained despite the appearance of early apoptotic features, including phosphatidylserine externalization, decreased mitochondrial membrane potential, cytochrome *c* release and high levels of caspase-3 activation. Notably, there was no finalization of PCD, manifested by DNA fragmentation, or the development of features of secondary necrosis. In this regard *S. aureus* clearly resembles *Legionella pneumophila*, an obligatory intracellular parasite of macrophages. The major difference between these two organisms is that *L. pneumophila* proliferates inside macrophages before lysing the host cells [21].

Induction of the apoptotic program by phagocytosed *S. aureus* appeared to be somewhat donor-dependent. Of the 25 individual donor-derived cultures of hMDMs, 12 cultures infected with *S. aureus* led to variable degrees of transient PCD activation. In all 12 cases the level of early apoptotic markers, including caspase-3 activity, peaked at 24 h post-phagocytosis and then returned to baseline levels. This indicates that cells from different individuals can respond differently to infection with *S. aureus*. It is likely that in nonresponsive cultures antiapoptotic mechanisms prevail from the outset of infection, while in responsive cells a balance between pro- and apoptotic pathways is established, protecting the infected macrophages from death. It would of great interest to determine if this difference translates into an altered, individual susceptibility to *S. aureus* infection.

The resistance of adherent macrophages to apoptosis is striking, particularly in context of the high susceptibility of circulating phagocytes (neutrophils and monocytes) to *S. aureus*-induced cell death [39]. In these cells the phagocytosis of *S. aureus* preferentially mediates cell death by the mitochondrial (intrinsic) apoptotic pathway, manifested by a rapid disruption of mitochondrial homeostasis, cytochrome *c* release, sequential activation of caspase-9 and -3 and consequential nuclear DNA fragmentation [17]. This suggests that the intrinsic signalling pathway of apoptosis is affected in the infected macrophages. Indeed, this was confirmed by showing that *S. aureus*-infected macrophages were protected from staurosporine-induced apoptosis, but not from cell death induced by activation of the extrinsic pathway *via* FAS receptor ligation on the cell surface.

The proapoptotic activity of staurosporine targets the mitochondria, causing release of cytochrome *c* and subsequent caspase-3 activation [40]. In infected macrophage cultures, staurosporine-induced release of cytochrome *c*, and the associated caspase activation, was significantly suppressed in comparison to mock-infected cells. This effect was observed regardless of the initial response of macrophages to *S. aureus* infection. Notably, protection was limited to a subset of cells which contain intracellular bacteria, whilst non-infected macrophages succumbed to apoptotic cell death. This means that the protective effect of *S. aureus* on STS-induced cell death, determined using whole cultures would be much higher if the assay was restricted only to those infected macrophages.

The observed cytoprotection was apparently *S. aureus*-specific, since engulfment of *B. subtilis*, *E. coli*, latex beads or prestimulation with phorbol ester (PMA) yielded practically no effect. These results exclude signaling pathways induced by the phagocytosis of inert particles (latex beads), PMA-activated kinases, and receptors engaged in the recognition and uptake of *B. subtilis* and *E. coli*, as being essential for protecting macrophages from STS-induced death. In the case of the partial protection yielded by phagocytosis of heat-killed *S. aureus* we hypothesize that this is due to the activation of macrophages by staphylococcal products (e.g., lipoteichoic acid or peptidoglycan), sensed by intracellular pattern recognition receptors (NODs) [41]. Moreover, lipoteichoic acids are known to delay the spontaneous apoptosis of neutrophils and increase their life-span *in vivo* [42]. Interestingly, the level of NOD2 transcript dramatically increased 24 h after *S. aureus* phagocytosis (data not shown) suggesting that NOD2 may contribute to sensing of the intracellular presence of *S. aureus* and contribute to the cytoprotective effect elucidated by phagocytosis of killed *S. aureus*. The most compelling observation, however, is the maximum level of protection yielded by the engulfment of live bacteria, which was significantly higher than that elicited by killed *S. aureus*. This indicates that the induced cytoprotection is the result of cross-talk between live, metabolitically active intracellular *S. aureus* and their macrophage host. In obligatory intracellular pathogens, including *Chlamydia* sp. and *Legionella pneumophila*, CPAF and SidF proteins, respectively, have been found as directly interfering with the execution of apoptosis. While CPAF exerts its cytoprotective effect by specifically stimulating the targeted degradation of the BH3 proapoptotic proteins, SidF neutralize Bcl-2 proteins with proapoptotic activities [43], [44]. Such factor(s), should they exist, have yet to be identified in *S. aureus*.

Our findings clearly indicate that *S. aureus* protects infected macrophages against STS-induced apoptotic cell death by preserving mitochondrial membrane potential and blocking cytochrome *c* release into the cytoplasm. The integrity of mitochondrial membranes is controlled by members of the Bcl-2/Bax protein family, with the balance between pro- and antiapoptotic members of these families playing an important role in determining the life-or-death decision of the cell [25]. Consistent with this dogma we find that *S. aureus* does not alter significantly the expression of Bax, a key proapoptotic member of the Bcl-2 family, in infected macrophages, but instead strongly upregulates the expression of *BCL2* and *MCL1* genes, which stabilize mitochondrial membrane potential.

Bcl-2 protects cells from a wide range of cytotoxic insults, including cytokine deprivation, UV and gamma irradiation, and chemotherapeutic drugs. This protein is largely membrane associated, and like its antiapoptotic homologues, can bind active BH3-only proteins, and as a result of this interaction likely prevent the release of cytochrome *c* from mitochondria [26], [27]. On the other hand, myeloid cell leukemia-1 protein (Mcl-1) can interact directly with the pro-apoptotic factors Bax and Bak, inhibiting their ability to initiate cell death [45]. This latter antiapoptotic pathway is exploited by *M. tuberculosis*, which induces resistance in infected macrophages by up-regulation of *MCL1* expression [46]. *MCL1* is an early-response gene, expressed during critical transitions in cell phenotype that can be rapidly induced and repressed [47] in contribution of many cell signalling pathways like JNK/STAT3, PIK3/AKT or NFκB [48]. As such, our data demonstrates that the kinetics of *MCL1* expression correlate with *S. aureus* survival. At the beginning of infection bacteria are eliminated very effectively (from 10^8^ to 10^7^ in first 24 h p.i.) [13]; apparently resulting from the initially effective mobilization of antimicrobial potential. At this time the highest level of *MCL1* expression was observed from the entire course of infection. In following days the amount of the *MCL1* transcript decreases in parallel with the diminishing numbers of viable intracellular *S. aureus*. Despite this decreased expression of *MCL1*, cytoprotection was sustained up to 4 days post *S. aureus* infection.

A recent study has shown that Mcl-1 is dispensable for macrophage survival *in vivo* [49]. Therefore, the involvement of alternative cytoprotective mechanism activated in *S. aureus*-infected macrophages is highly probable. In keeping with this, our microarray analysis of gene expression revealed that *S. aureus* infection caused up-regulation of 14 antiapoptotic genes, and down-regulation of transcripts encoding 2 proapoptotic determinants. Notably, the expression of several proapoptotic genes was also upregulated. However, the increased expression of these genes was observed predominantly at 8 h, whereas the upregulation of antiapoptotic genes was sustained for up to 48 h. Finally, the level of transcripts of few genes with mixed pro- and antiapoptotic functions were elevated in infected macrophages, but their effect on cell survival in our experimental model was not determined. Therefore, we proposed a model whereby *S. aureus* alters the profile of host gene expression in infected macrophages, leading to an inhibition of apoptosis. In this model signaling *via* pattern recognition receptors, which activate the NFκB transcriptional factor, may explain the partial cytoprotection induced by heat-killed *S. aureus*. These conclusions are strongly supported by the notion that several intracellular pathogens induce a resistance to apoptosis within infected cells through robust alterations in gene expression patterns [41], [42], [50], [51]. This is most certainly the case for *C. pneumoniae*, which mediated this process via activation of NFκB [52]. In our study we also observed activation of NFκB shortly after *S. aureus* phagocytosis (data not shown). However, it needs to be investigated weather changes in other genes expression were dependent on the NFκB activity.

Taken together, the modulation of apoptosis in human macrophages by *S. aureus* seems to be a novel occurrence. During the early stages of infection *S. aureus* induces strong caspase-3 activation, whilst also triggering robust antiapoptotic mechanism(s). This latter event is associated with the down-regulation of proapoptotic genes and significant up-regulation of antiapoptotic genes, especially those preserving the potential of mitochondrial membrane and blocking cytochrome *c* release. Rigorous antiapoptotic signaling cascades apparently counteract the effect of caspase-3 activation, and efficiently prevent activation of the intrinsic, mitochondria-dependent pathway of cell death, rendering them refractory to potent stimulators of the intrinsic apoptotic pathway. As *S. aureus* induces apoptosis in human monocytes and neutrophils [17], [39], as well other eukaryotic cells [8], [9], it seems that activation of the antiapoptotic machinery is macrophage-specific. Although the precise mechanisms of antiapoptotic control mediated by *S. aureus* (from the standpoint of the bacterial factors involved) remain to be elucidated, our findings seem to have significant implications for staphylococcal pathogenicity. The inhibition of apoptosis in macrophages infected with the *S. aureus* Newman strain resistant to intracellular killing [13] prolongs the integrity of these mobile cells, and may significantly contribute to pathogen dissemination and the chronic character of some staphylococcal infections. Conversely, the resilience of infected macrophages to pathogen-induced damage may be essential for regulating inflammation and immune responses to *S. aureus*. Thus it is clear that a deeper understanding of how *S. aureus* modulates the functions of macrophages will be of prime importance to the development of novel therapeutic interventions, and other mechanisms by which to control diseases caused by this bacterium.

## References

[1] Archer GL (1998). *Staphylococus aureus*: A well-armed pathogen.. Clin Infect Dis.

[2] Lowy FD (1998). *Staphylococcus aureus* infections.. N Engl J Med.

[3] Potempa J, Pike RN (2009). Corruption of innate immunity by bacterial proteases.. J Innate Immunity.

[4] Zetola N, Francis JS, Nuermberger EL, Bishai WR (2005). Community-acquired meticillin-resistant *Staphylococcus aureus*: an emerging threat.. Lancet Infect Dis.

[5] Liñares J (2001). The VISA/GISA problem: therapeutic implication.. Clin Microbiol Infect.

[6] Hiramatsu K (2001). Vancomycin-resistant *Staphylococcus aureus*: a new model of antibiotic resistance.. Lancet Infect Dis.

[7] Hudson MC, Ramp WK, Nicholson NC, Williams AS, Nousiainen MT (1995). Internalization of *Staphylococcus aureus* by cultured osteoblasts.. Microb Pathog.

[8] Bayles KW, Wesson CA, Liou LE, Fox LK, Bohach GA (1998). Intracellular *Staphylococcus aureus* escapes the endosome and induces apoptosis in epithelial cells.. Infect Immun.

[9] Menzies BE, Kourteva I (1998). Internalization of *Staphylococcus aureus* by endothelial cells induces apoptosis.. Infect Immun.

[10] Lowy FD (2000). Is *Staphylococcus aureus* an intracellular pathogen?. Trends Microbiol.

[11] Rogers DE, Tompsett R (1952). The survival of staphylococci within human leukocytes.. J Exp Med.

[12] Voyich JM, Braughton KR, Sturdevant DE, Whitney AR, Saïd-Salim B (2005). Insights into mechanisms used by *Staphylococcus aureus* to avoid destruction by human neutrophils.. J Immunol.

[13] Kubica M, Guzik K, Koziel J, Zarebski M, Richter W (2008). A potential new pathway for *Staphylococcus aureus* dissemination: the silent survival of *S. aureus* phagocytosed by human monocyte-derived macrophages.. PLoS ONE.

[14] Zychlinsky A, Prevost MC, Sansonetti PJ (1992). *Shigella flexneri* induces apoptosis in infected macrophages.. Nature.

[15] Ameisen JC, Estaquier J, Idziorek T (1994). From AIDS to parasite infection: pathogen-mediated subversion of programmed cell death as a mechanism for immune dysregulation.. Immunol Rev.

[16] Baran J, Guzik K, Hryniewicz W, Ernst M, Flad HD (1996). Apoptosis of monocytes and prolonged survival of granulocytes as a result of phagocytosis of bacteria.. Infect Immun.

[17] Lundqvist-Gustafsson H, Norrman S, Nilsson J, Wilsson A (2001). Involvement of p38-mitogen-activated protein kinase in *Staphylococcus aureus*-induced neutrophil apoptosis.. J Leukoc Biol.

[18] Smagur J, Guzik K, Magiera L, Bzowska M, Gruca M (2009). A new pathway of staphylococcal pathogenesis: Apoptosis-like death induced by Staphopain B (SspB) in human peripheral blood neutrophils and monocytes.. J Innate Immun.

[19] Williams GT (1994). Programmed cell death: a fundamental protective response to pathogens.. Trends Microbiol.

[20] Akarid K, Arnoult D, Micic-Polianski J, Sif J, Estaquier J (2004). *Leishmania major*-mediated prevention of programmed cell death induction in infected macrophages is associated with the repression of mitochondrial release of cytochrome c.. J Leukoc Biol.

[21] Abu-Zant A, Santic M, Molmeret M, Jones S, Helbig J (2005). Incomplete activation of macrophage apoptosis during intracellular replication of *Legionella pneumophila*.. Infect Immun.

[22] Fischer SF, Schwarz C, Vier J, Häcker G (2001). Characterization of antiapoptotic activities of *Chlamydia pneumoniae* in human cells.. Infect Immun.

[23] Häcker G, Kirschnek S, Fischer SF (2006). Apoptosis in infectious disease: how bacteria interfere with the apoptotic apparatus.. Med Microbiol Immunol.

[24] Ashkenazi A, Dixit VM (1998). Death receptors: signaling and modulation.. Science.

[25] Green DR, Reed JC (1998). Mitochondria and apoptosis.. Science.

[26] Cory S, Adams JM (2002). The Bcl2 family: regulators of the cellular life-or-death switch.. Nat Rev Cancer.

[27] Yang J, Liu X, Bhalla K, Kim CN, Ibrado AM (1997). Prevention of apoptosis by Bcl-2: release of cytochrome c from mitochondria blocked.. Science.

[28] Jürgensmeier JM, Xie Z, Deveraux Q, Ellerby L, Bredesen D (1998). Bax directly induces release of cytochrome c from isolated mitochondria.. Proc Natl Acad Sci U S A.

[29] Boldrick JC, Alizadeh AA, Diehn M, Dudoit S, Liu CL (2002). Stereotyped and specific gene expression programs in human innate immune responses to bacteria.. Proc Natl Acad Sci U S A.

[30] Nau GJ, Richmond JF, Schlesinger A, Jennings EG, Lander ES (2002). Human macrophage activation programs induced by bacterial pathogens.. Proc Natl Acad Sci U S A.

[31] Holland SM, DeLeo FR, Elloumi HZ, Hsu AP, Uzel G (2007). STAT3 mutations in the hyper-IgE syndrome.. N Engl J Med.

[32] Livak KJ, Schmittgen TD (2001). Analysis of relative gene expression data using real-time quantitative PCR and the 2(-Delta Delta C(T)) method.. Methods.

[33] Lee SY, Cherla RP, Tesh VL (2007). Simultaneous induction of apoptotic and survival signaling pathways in macrophage-like THP-1 cells by Shiga toxin 1.. Infect Immun.

[34] Okuda J, Arikawa Y, Takeuchi Y, Mahmoud MM, Suzaki E (2006). Intracellular replication of *Edwardsiella tarda* in murine macrophage is dependent on the type III secretion system and indicates an up-regulation of anti-apoptotic NF-kappaB target genes protecting the macrophages from staurosporine-induced apoptosis.. Microb Pathog.

[35] Desagher S, Martinou JC (2000). Mitochondria as the central control point of apoptosis.. Trends Cell Biol.

[36] Hiroi M, Shimojima T, Kashimata M, Miyata T, Takano H (1998). Inhibition by *Porphyromonas gingivalis* LPS of apoptosis induction in human peripheral blood polymorphonuclear leukocytes.. Anticancer Res.

[37] Shirin H, Sordillo EM, Kolevska TK, Hibshoosh H, Kawabata Y (2000). Chronic *Helicobacter pylori* infection induces an apoptosis-resistant phenotype associated with decreased expression of p27(kip1).. Infect Immun.

[38] Nakhjiri SF, Park Y, Yilmaz O, Chung WO, Watanabe K (2001). Inhibition of epithelial cell apoptosis by *Porphyromonas gingivalis*.. FEMS Microbiol Lett.

[39] Kobayashi SD, Braughton KR, Whitney AR, Voyich JM, Schwan TG (2003). Bacterial pathogens modulate an apoptosis differentiation program in human neutrophils.. Proc Natl Acad Sci U S A.

[40] Weil M, Jacobson MD, Coles HS, Davies TJ, Gardner RL (1996). Constitutive expression of the machinery for programmed cell death.. J Cell Biol.

[41] Leber JH, Crimmins GT, Raghavan S, Meyer-Morse NP, Cox JS (2008). Distinct TLR- and NLR-mediated transcriptional responses to an intracellular pathogen.. Plos Pathogens.

[42] Lotz S, Aga E, Wilde I, van Zandbergen G, Hartung T (2004). Highly purified lipoteichoic acid activates neutrophil granulocytes and delays their spontaneous apoptosis via CD14 and TLR2.. J Leukoc Biol.

[43] Ying S, Seiffert BM, Häcker G, Fischer SF (2005). Broad degradation of proapoptotic proteins with the conserved Bcl-2 homology domain 3 during infection with *Chlamydia trachomatis*.. Infect Immun.

[44] Banga S, Gao P, Shen X, Fiscus V, Zong WX (2007). *Legionella pneumophila* inhibits macrophage apoptosis by targeting pro-death members of the Bcl2 protein family.. Proc Natl Acad Sci U S A.

[45] Leu JI, Dumont P, Hafey M, Murphy ME, George DL (2004). Mitochondrial p53 activates Bak and causes disruption of a Bak-Mcl1 complex.. Nat Cell Biol.

[46] Sly LM, Hingley-Wilson SM, Reiner NE, McMaster WR (2003). Survival of *Mycobacterium tuberculosis* in host macrophages involves resistance to apoptosis dependent upon induction of antiapoptotic Bcl-2 family member Mcl-1.. J Immunol.

[47] Yang T, Buchan HL, Townsend KJ, Craig RW (1996). MCL-1, a member of the BCL-2 family, is induced rapidly in response to signals for cell differentiation or death, but not to signals for cell proliferation.. J Cell Physiol.

[48] Liu H, Perlman H, Pagliari LJ, Pope RM (2001). Constitutively activated Akt-1 is vital for the survival of human monocyte-differentiated macrophages: Role of Mcl-1, independent of Nuclear Factor (NF)-B, Bad, or caspase activation.. J Exp Med.

[49] Dzhagalov I, St John A, He YW (2007). The antiapoptotic protein Mcl-1 is essential for the survival of neutrophils but not macrophages.. Blood.

[50] Binnicker MJ, Williams RD, Apicella MA (2004). Gonococcal porin IB activates NF-kappaB in human urethral epithelium and increases the expression of host antiapoptotic factors.. Infect Immun.

[51] Joshi SG, Francis CW, Silverman DJ, Sahni SK (2004). NF-kappaB activation suppresses host cell apoptosis during *Rickettsia rickettsii* infection via regulatory effects on intracellular localization or levels of apoptogenic and anti-apoptotic proteins.. FEMS Microbiol Lett.

[52] Wahl C, Oswald F, Simnacher U, Weiss S, Marre R (2001). Survival of *Chlamydia pneumoniae*-infected Mono Mac 6 cells is dependent on NF-kappaB binding activity.. Infect Immun.

[53] Hu X, Yee E, Harlan JM, Wong F, Karsan A (1998). Lipopolysaccharide induces the antiapoptotic molecules, A1 and A20, in microvascular endothelial cells.. Blood.

[54] Tsubata T, Wu J, Honjo T (1993). B-cell apoptosis induced by antigen receptor crosslinking is blocked by a T-cell signal through CD40.. Nature.

[55] Irmler M, Thome M, Hahne M, Schneider P, Hofmann K (1997). Inhibition of death receptor signals by cellular FLIP.. Nature.

[56] Fujisawa A, Kambe N, Saito M, Nishikomori R, Tanizaki H (2007). Disease-associated mutations in CIAS1 induce cathepsin B-dependent rapid cell death of human THP-1 monocytic cells.. Blood.

[57] Keita M, Leblanc C, Andrews D, Ramanathan S (2007). GIMAP5 regulates mitochondrial integrity from a distinct subcellular compartment.. Biochem Biophys Res Commun.

[58] Bernal-Mizrachi L, Lovly CM, Ratner L (2006). The role of NFκB-1 and NFκB-2-mediated resistance to apoptosis in lymphomas.. Proc Natl Acad Sci U S A.

[59] Katoh O, Tauchi H, Kawaishi K, Kimura A, Satow Y (1995). Expression of the vascular endothelial growth factor (VEGF) receptor gene, KDR, in hematopoietic cells and inhibitory effect of VEGF on apoptotic cell death caused by ionizing radiation.. Cancer Res.

[60] Zhou P, Qian L, Kozopas KM, Craig RW (1997). Mcl-1, a Bcl-2 family member, delays the death of hematopoietic cells under a variety of apoptosis-inducing conditions.. Blood.

[61] Rebollo A, Dumoutier L, Renauld JC, Zaballos A, Ayllón V (2000). Bcl-3 expression promotes cell survival following interleukin-4 deprivation and is controlled by AP1 and AP1-like transcription factors.. Mol Cell Biol.

[62] Battle TE, Frank DA (2002). The role of STATs in apoptosis.. Curr Mol Med.

[63] Conte D, Holcik M, Lefebvre CA, Lacasse E, Picketts DJ (2006). Inhibitor of apoptosis protein cIAP2 is essential for lipopolysaccharide-induced macrophage survival.. Mol Cell Biol.

[64] Lee NK, Lee SY (2002). Modulation of life and death by the tumor necrosis factor receptor-associated factors (TRAFs).. J Biochem Mol Bio.

[65] Freeman SN, Ma Y, Cress WD (2008). RhoBTB2 (DBC2) is a mitotic E2F1 target gene with a novel role in apoptosis.. J Biol Chem.

[66] Wajant H (2003). Death receptors.. Essays Biochem.

[67] Monack DM, Navarre WW, Falkow S (2001). *Salmonella*-induced macrophage death: the role of caspase-1 in death and inflammation.. Microbes Infect.

[68] Droin N, Dubrez L, Eymin B, Renvoizé C, Bréard J (1998). Upregulation of CASP genes in human tumor cells undergoing etoposide-induced apoptosis.. Oncogene.

[69] Bender LM, Morgan MJ, Thomas LR, Liu ZG, Thorburn A (2005). The adaptor protein TRADD activates distinct mechanisms of apoptosis from the nucleus and the cytoplasm.. Cell Death Differ.

[70] Mangan DF, Welch GR, Wahl SM (1991). Lipopolysaccharide, tumor necrosis factor-alpha, and IL-1 beta prevent programmed cell death (apoptosis) in human peripheral blood monocytes.. J Immunol.

[71] Goepel F, Weinmann P, Schymeinsky J, Walzog B (2004). Identification of caspase-10 in human neutrophils and its role in spontaneous apoptosis.. J Leukoc Biol.

[72] Shikama Y, Yamada M, Miyashita T (2003). Caspase-8 and caspase-10 activate NF-kappaB through RIP, NIK and IKKalpha kinases.. Eur J Immunol.

